# A Programmable Mechanical Maxwell’s Demon

**DOI:** 10.3390/e21010065

**Published:** 2019-01-14

**Authors:** Zhiyue Lu, Christopher Jarzynski

**Affiliations:** 1 James Franck Institute, University of Chicago, Chicago, IL 60637, USA; 2 Institute for Physical Science and Technology, University of Maryland, College Park, MD 20742, USA; 3 Department of Chemistry and Biochemistry, University of Maryland, College Park, MD 20742, USA; 4 Department of Physics, University of Maryland, College Park, MD 20742, USA

**Keywords:** Maxwell’s demon, Shannon entropy, information engine, Landauer’s principle, Szilard engine, second law of thermodynamics

## Abstract

We introduce and investigate a simple and explicitly mechanical model of Maxwell’s demon—a device that interacts with a memory register (a stream of bits), a thermal reservoir (an ideal gas) and a work reservoir (a mass that can be lifted or lowered). Our device is similar to one that we have briefly described elsewhere, but it has the additional feature that it can be programmed to recognize a chosen reference sequence, for instance, the binary representation of π. If the bits in the memory register match those of the reference sequence, then the device extracts heat from the thermal reservoir and converts it into work to lift a small mass. Conversely, the device can operate as a generalized Landauer’s eraser (or copier), harnessing the energy of a dropping mass to write the chosen reference sequence onto the memory register, replacing whatever information may previously have been stored there. Our model can be interpreted either as a machine that autonomously performs a conversion between information and energy, or else as a feedback-controlled device that is operated by an external agent. We derive generalized second laws of thermodynamics for both pictures. We illustrate our model with numerical simulations, as well as analytical calculations in a particular, exactly solvable limit.

## 1. Introduction

The field of information thermodynamics traces its origins to a whimsical, 150-year-old thought experiment. In a letter to a friend [1], James Clerk Maxwell introduced a hypothetical “neat-fingered being”, now universally known as *Maxwell’s demon*, who brings about an apparent violation of the second law of thermodynamics, simply by observing the motions of gas molecules and manipulating a trapdoor to segregate faster from slower molecules. While Maxwell emphasized the role of the demon’s intelligence, subsequent researchers—notably including Marian Smoluchowski [2] and Richard Feynman [3]—have considered whether a dumb device might be able to accomplish similar results, and if so, what the existence of such a device would imply about the status of the second law. In recent decades, a consensus has formed around a perspective developed largely by Rolf Landauer, Oliver Penrose and Charles Bennett [4,5,6]. At the heart of this perspective is the notion that, if Maxwell’s demon were a purely physical machine, then the information it gathers must be stored in a physical memory register, commonly represented as a sequence of classical bits. The writing of this information increases the entropy of the bits, thereby (so the argument goes) compensating for the decrease of entropy that occurs elsewhere as the machine “violates” the second law. Bennett’s analysis of chemical proofreading [7] provides an early model system illustrating this idea.

The past decade has seen renewed interest in this topic, motivated in part by its connections with fluctuation theorems and related advances in non-equilibrium statistical physics [8,9,10], as well as by improved experimental capabilities for manipulating small systems [11,12,13,14,15,16,17,18,19,20,21,22,23,24]. Progress has included sharpened relationships between thermodynamic and information-theoretic quantities [25,26,27,28,29,30,31,32,33] as well as a variety of simple model systems more explicit than those explored in the past [17,18,19,22,34,35,36,37,38,39,40,41,42,43,44,45,46,47,48].

Two broad paradigms have emerged in these investigations—*autonomous* and *non-autonomous* demons. The non-autonomous paradigm echoes Maxwell’s original idea: an external agent that is in a sense “outside of Physics” (the demon) performs feedback control on a material object (e.g., a trapdoor) to accomplish a task apparently prohibited by the second law. This task may be the creation of a temperature gradient as in Maxwell’s scenario, or the conversion of heat into work as in many later models such as the Szilard engine [35]. The key idea is that the agent rectifies thermal fluctuations, using the information it gains by observing nanoscale motions. In this paradigm, the thermodynamic benefits delivered by the agent—such as work generated to lift a mass against gravity—are related to the amount of information it gathers about its surroundings.

By contrast, the autonomous paradigm is all-inclusive, in that the demon and, importantly, its memory are explicitly modeled as physical systems [34,39,42,43,45,46,47,48]. In this paradigm, the goal is often to illustrate how a physical machine might actually accomplish results similar to those of Maxwell’s imagined neat-fingered being, and to explore quantitatively how the thermodynamic benefits that the machine delivers are related to changes in the information content of its memory.

**Figure 1:**
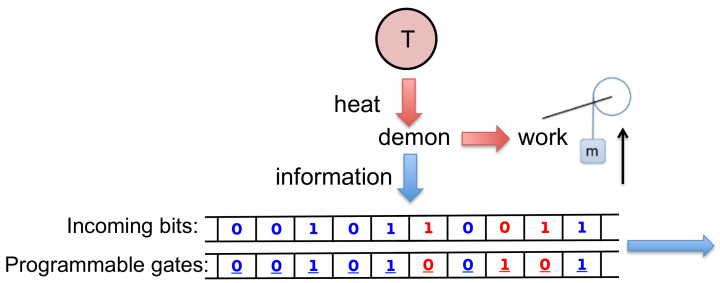
In our schematic conception of a programmable, autonomous Maxwell’s demon, a fixed set of binary gates defines a *reference sequence*. As the demon interacts one bit at a time with an incoming sequence of memory bits, it is able to lift a small mass against gravity if the incoming bit sequence matches the reference sequence. As the demon writes information onto the memory bits, the outgoing sequence becomes less correlated with the reference sequence. To highlight the correlation between each bit–gate pair, we use blue when the pair are in the same state and red when the pair are in the opposite state. Conversely, if the mass is large and falls against gravity, then this energy can be used to copy the reference sequence to the memory bits.

**Figure 2:**
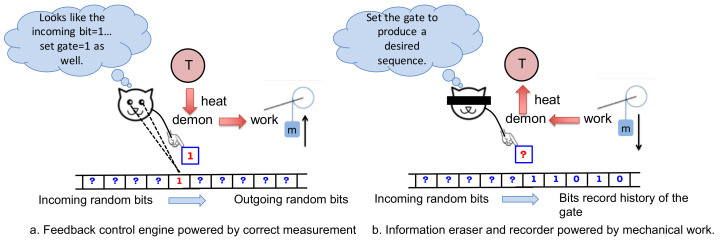
Alternatively, our model can illustrate a non-autonomous device operated via feedback control by an external agent. (**a**) In the engine mode, which resembles Szilard’s thought experiment [35], the agent measures each incoming memory bit and switches a gate accordingly. When these measurements are accurate, the procedure induces a bias toward counter-clockwise rotation that can be harnessed to lift a small mass against gravity; (**b**) If the mass is large and falls against gravity, the energy that is released can be used to write a sequence chosen by the agent, onto the outgoing bit stream. In this mode, the agent does not measure the incoming bits, but rather manipulates the gate to encode the desired sequence.

**Figure 3:**
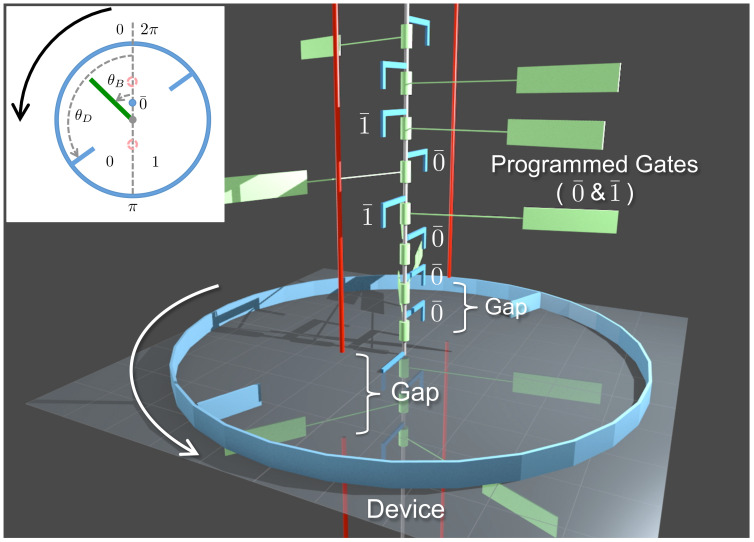
Snapshot of our programmable demon. A series of green paddles move down frictionlessly along the central axle. The paddles are separated by the red bars into binary states, left (0) and right (1)—see inset. Each bit passes by the rotational ring (the blue ring with two inward blades) for the same finite amount of time, during which it can change states. We claim that if the incoming bits (000101⋯) are in agreement with the programmed gates ($\bar{0}\bar{0}\bar{0}\bar{1}\bar{0}\bar{1}\cdots$), then the ring favors CCW motion, which can be used to lift an external load. A top view of the system is shown in the inset. A video clip illustrating the dynamics of our demon is found at https://youtu.be/LkYljJ__-Cs.

In the present paper, we introduce and analyze a model of Maxwell’s demon that can be interpreted within either the autonomous or the non-autonomous paradigm (Figure 1 and Figure 2). Our model builds on one that we briefly described, with our colleague Dibyendu Mandal, in 2014 [34]. Unlike earlier models involving systems making stochastic transitions among a discrete set of states [39,40,42,43,44,45,47,48], our model is entirely mechanistic—the demon and its memory consist of frictionless, moving components immersed in a dilute gas, evolving under Newtonian dynamics. Specifically, the demon is a rotational ring equipped with two blades and the memory is represented by a sequence of rotating paddles, as shown in Figure 3 and discussed in greater detail in Section 2. We showed in Ref. [34] that, if the system’s memory is initialized in a “clean” state corresponding to the bit sequence “…00000…”, then the mechanistic interplay between the ring, the paddles and the dilute gas produces rotational motion that lifts a small mass against gravity. In this mode of operation, the entire contraption is an *information engine*, rectifying thermal fluctuations to convert heat into work—the fuel for this process is provided by the randomization of the memory, as the clean bit stream is converted to a “polluted” mixture of 0s and 1s. Conversely, if the memory begins in a random mixture “…01101…”, then a large mass that drops with gravity can be harnessed to reset all the bits to 0 s, illustrating Landauer’s principle [4] that work is required to erase information.

In Ref. [34], a clean memory register is equated with the uniform bit sequence “…00000…”. In principle, however, what matters is not uniformity but rather lack of randomness, as quantified by Shannon entropy. Let us use the term *generalized clean memory* to denote an arbitrary but *fully determined* bit sequence, for instance the binary representation of π. Since a fully determined sequence is entropically equivalent to the sequence “…00000…”, a generalized clean memory should be able to serve as a thermodynamic resource to drive an information engine. This consideration motivates us to design a mechanical information engine that operates on a generalized clean memory. Our model is programmable, in the following sense: for any choice of pre-determined *reference sequence*—be it the binary representation of π, or the repeating sequence “…010101…”, or for that matter the uniform sequence “…000000…”—we can program the system so that, if the memory bits are initialized in this reference sequence, then the machine operates as an information engine, lifting a small mass against gravity, as illustrated schematically in Figure 1. Conversely, if the bits are initialized in a different sequence, then the energy from a falling large mass can be used to write the reference sequence onto the bits (rather than resetting them all to the 0 state as in Ref. [34]).

As we describe in further detail, our system is programmed using a sequence of binary *programmable gates*. Notice that here we use the term “gate” to denote a physical object that blocks the motion of a paddle (see Figure 3), rather than to denote the concept of a logical gate. If these gates are fixed, prior to the start of the process, to match the chosen reference sequence, then the machine operates as an autonomous Maxwell demon. However, we can alternatively imagine that an external agent arranges the gates on the fly, one by one, using information based on real-time observations. By thus reinterpreting each programmable gate as a binary switch that is feedback controlled by the agent, our model becomes an illustration of a non-autonomous Maxwell demon. Analogous to the autonomous picture, where the system can operate in either an engine mode or an information copier mode, the agent-involved feedback control picture operates either as an engine or information recorder (see Figure 2).

The paper is structured as follows. In Section 2, we describe the various components of our device, and we sketch how it can operate as an autonomous information engine. In Section 3, we describe in detail the three possible modes of operation of our autonomous device: as an engine, an eraser (or copier) and a “dud”. In Section 4, we illustrate these modes of operation using numerical simulations, we solve explicitly for the behavior of the model in a particular “slow-moving” limit, and we consider its thermodynamic description, including its efficiency. In Section 5, we discuss how our model can be used to illustrate a non-autonomous machine, operated by an external agent using measurement and feedback—as in Maxwell’s original thought experiment—and we obtain a bound on the amount of work that this machine can deliver. We end in Section 6 with a brief summary and discussion.

## 2. Programmable Maxwell’s Demon

### 2.1. Components and Basic Design

As mentioned, the machine described in this paper is equipped with a binary reference sequence that can be preprogrammed to any desired pattern of 0s and 1s—for instance, the binary representation of π. This *reference sequence* is fixed, and is distinct from the sequence of *memory bits* that interact dynamically with the rotational ring. We argue that, if the incoming memory bits match the reference sequence, then the ring favors counter-clockwise (CCW) rotation that can be used to perform work against an external load.

As illustrated in Figure 3, the entire machine consists of three components—a sequence of paddles acting as the bits of a memory register, a set of fixed gates that encode a preprogrammed binary reference sequence, and the demon that is realized by a rotational ring: a ring that interacts with the memory register via blade–paddle collision and can perform work via rotation against a constant external force. We now describe these components in detail, beginning with the paddles that constitute the bits of the memory register. These paddles rotate frictionlessly around a central axle. The orientation of a paddle is given by an angle $\theta_{B}$. When $\theta_{B}\in \left(0,\pi \right)$, the paddle represents a bit in the 0 state, and, when $\theta_{B}\in \left(\pi ,2\pi \right)$, it represents a bit in the 1 state. Two blocking bars (shown as vertical red bars) located at angles 0 and π prevent each bit from spontaneously flipping between the 0 and 1 states. Each blocking bar contains a gap, as shown in Figure 3. The central axle moves downward at a constant speed, carrying the bits and gates past the demon. The entire machine is immersed in an ideal gas in thermal equilibrium at temperature *T*. The gas particles collide elastically with the paddles, causing them to undergo Brownian-like rotation around the axle. For clarity, the gas particles are not shown in the figure.

The preprogrammed reference sequence is encoded in a set of rigid gates attached to the central axle, which accompany the paddles as they move downward past the demon. These gates are shown as L-shaped blue bars extending perpendicularly from the axle. The orientation of a gate is fixed at either θ=0 (representing state $\bar{0}$) or θ=π (state $\bar{1}$). When a paddle and its gate arrive at the vertical location of the gaps on the red bars, the paddle is able to switch its state by passing through the gap that is not blocked by the gate. For example, if the gate is in state $\bar{0}$, the gap at θ=0 is blocked, and the bit paddle can switch its state by passing through the gap at θ=π.

The rigid ring is equipped with two inward-pointing blades, attached at opposite locations. The ring rotates freely around the central axle but does not translate or wobble. The angular orientation of the ring is specified by $\theta_{D}$; see inset of Figure 3. Similar to the paddles, the ring undergoes Brownian-like rotation due to elastic collisions between its blades and the gas particles. Additionally, the ring’s blades can collide elastically with the paddles as they move past it. The ring is situated at the vertical height of the gaps in the blocking bars. The spacing between bits, the size of the gaps, and the vertical widths of the paddles and the ring’s blades are set so that, at any time, there is exactly one paddle within the vertical range of the gap, and that paddle is simultaneously within the vertical collision range of the ring’s blades. This paddle is called the *interacting bit*, and its gate is called the *engaged gate*. We use the term *interaction interval* to denote the interval of time during which a given paddle acts as an interacting bit, and its gate acts as the engaged gate. The duration of the interaction interval, $\tau^{\mathrm{int}}$, is the same for each paddle and gate.

The life cycle of a given paddle (memory bit) then proceeds as follows. Prior to arriving at the vertical level of the ring, the orientation of the paddle, $\theta_{B}$, performs Brownian-like motion but the binary state of the bit (0 or 1) is frozen due to the presence of the blocking bars. This binary state represents an *incoming* memory bit. Then, over the course of an interaction interval of duration $\tau^{\mathrm{int}}$, the paddle can switch between the 0 and 1 states, by passing through the gap that is not blocked by the reference gate; during this interval, the paddle also interacts with the blades of the ring. Finally, after the interaction interval, as the paddle passes below the vertical level of the ring, the binary state of the paddle is once again frozen due to the blocking bars—at this point, the paddle represents an *outgoing* memory bit.

### 2.2. Memory Register—A Sequence of Bits

The binary state of an incoming memory bit (paddle), b∈{0,1}, might or might not be the same as the binary state of the corresponding reference bit (gate), $g\in \{\bar{0},\bar{1}\}$. We characterize the cleanness of the incoming bit sequence $\left(\cdots b_{n-1},b_{n},b_{n+1}\cdots \right)$ by the degree to which it matches the fixed reference sequence $\left(\cdots g_{n-1},g_{n},g_{n+1}\cdots \right)$. If the binary state of each incoming memory bit matches that of the accompanying gate, i.e., if $b_{n}=g_{n}\forall n$, then the memory is considered to be perfectly clean. If the incoming sequence contains mismatches between memory and reference bits, then these mismatches are considered to be impurities that pollute the memory sequence.

Let $P_{\mathrm{in}}\left(\mathrm{same}\right)$ denote the fraction of incoming bits that are correctly matched with their reference bits ($0\bar{0}$ or $1\bar{1}$), and $P_{\mathrm{in}}\left(\mathrm{diff}\right)$ the fraction that are mismatched ($0\bar{1}$ or $1\bar{0}$). We assume that the probability of a mismatch is independent of the state of the reference bit, and the mismatches are statistically uncorrelated with one another. We quantify the cleanness of the incoming memory by the excess ratio of clean bits:

(1) $$\delta =P_{\mathrm{in}}\left(\mathrm{same}\right)-P_{\mathrm{in}}\left(\mathrm{diff}\right)\in \left[-1,+1\right]$$

It is useful at this point to introduce a logical variable *L* that is the *Boolean equality* between the states of the bit and the gate: L=B Exy G [49]. That is, the value of *L* is given by

(2) $$\begin{array}{c} l=\mathrm{true}\equiv \mathrm{same}\;\mathrm{if}\;b=g\\ l=\mathrm{false}\equiv \mathrm{diff}\;\;\;\mathrm{if}\;b\neq g \end{array}$$

Here and below, we use the capital letters *B*, *G* and *L* to refer to binary variables, and lower case *b*, *g* and *l* to denote the values of these variables. The sequences of incoming memory and reference bits together specify a sequence $\left(\cdots l_{n-1},l_{n},l_{n+1}\cdots \right)$, whose Shannon entropy, per bit, is given by

(3) $$S_{L,\mathrm{in}}=-\left[P_{\mathrm{in}}\left(\mathrm{same}\right)\log P_{\mathrm{in}}\left(\mathrm{same}\right)+P_{\mathrm{in}}\left(\mathrm{diff}\right)\log P_{\mathrm{in}}\left(\mathrm{diff}\right)\right]\in \left[0,\log2\right]$$

For the outgoing bits $\left(\cdots b_{n-1}^{\prime },b_{n}^{\prime },b_{n+1}^{\prime }\cdots \right)$, we can similarly define $P_{\mathrm{out}}\left(\mathrm{same}\right)$, $P_{\mathrm{out}}\left(\mathrm{diff}\right)$ and

(4) $$S_{L,\mathrm{out}}=-\left[P_{\mathrm{out}}\left(\mathrm{same}\right)\log P_{\mathrm{out}}\left(\mathrm{same}\right)+P_{\mathrm{out}}\left(\mathrm{diff}\right)\log P_{\mathrm{out}}\left(\mathrm{diff}\right)\right]$$

The difference $\Delta S_{L}=S_{L,\mathrm{out}}-S_{L,\mathrm{in}}$ quantifies the cleanness of the memory sequence, per bit, due to the interactions between the memory bits and ring. While the interaction between the memory bits and the demon might in principle induce correlations among the outgoing bits, in our analysis, we ignore these correlations.

### 2.3. Work Reservoir—A Mass that Can Be Raised or Lowered

In addition to the elements described above, an external load, Γ, exerts a constant torque on the ring that is positive when the torque favors rotation in the clockwise (CW) direction. This load is generated by a mass that hangs from a string wrapped around the ring—the gravitational force on the mass produces a CW torque on the ring. If the ring rotates in the counter-clockwise (CCW) direction, the mass is lifted upwards. This mass is not shown in Figure 3.

**Figure 4:**
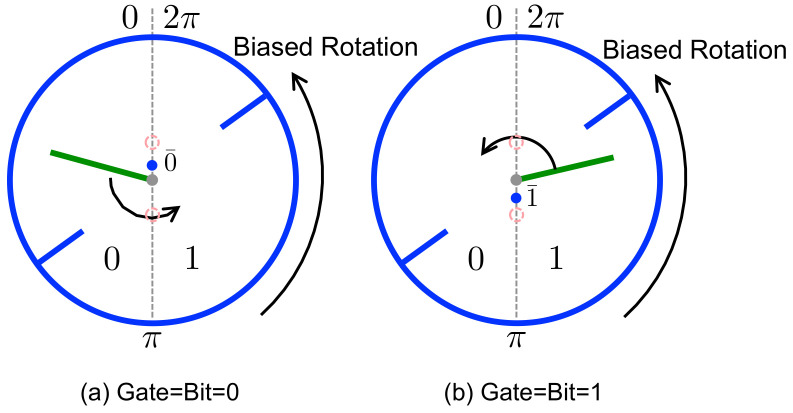
Engine Mode. The ring prefers CCW rotation when the bit starts with the state that is in agreement with its corresponding gate. The blue dots represent the programmed gates.

It is useful to understand the operation of our machine in the absence of this load, i.e., when Γ=0. To that end, let us first assume that the incoming bit sequence is perfectly clean: the binary state of each memory bit matches that of its reference bit (δ=+1). There are then two possible combinations for an incoming memory and reference bit, $\left(0\bar{0}\right)$ and $\left(1\bar{1}\right)$, as illustrated in Figure 4. In the former case (Figure 4a), the paddle is initially confined (by the blocking bars) within the angular range $\theta_{B}\in \left(0,\pi \right)$. During the interaction interval, this paddle has the opportunity to “expand” into the full circular range (0,2π), by swinging through the gap located at θ=π. This opportunity produces a statistical bias that favors CCW rotation, which in turn induces a CCW rotational bias for the ring, due to the possibility of collisions between the paddle and the ring’s blades. For the incoming combination $\left(1\bar{1}\right)$, the expansion of the memory bit during the interaction again interval favors CCW rotation as the reference gate blocks the gap at θ=π (Figure 4b). In this manner, over the course of many interaction intervals, the ring settles into a steady state in which the ring rotates systematically in the counterclockwise direction—the thermal fluctuations generated by collisions with the gas particles are rectified to produce directed rotation. In this steady state, there is a continual exchange of energy (due to collisions) between the ring’s blades and the gas, but this exchange does not lead to a net flow of energy in one direction.

By similar arguments, the maximally unclean situation (δ=−1) produces an identical bias in the clockwise direction. More generally, each correctly matched pair of memory and reference bits generates a bias toward CCW rotation, while each mismatched pair generates a bias toward CW rotation. Hence, over many interaction intervals, our ring (in the absence of an external load) produces a rotational bias whose direction is CCW for δ>0 and CW for δ<0. The strength of the bias is quantified by |δ|.

Let us now assume δ>0 and imagine that we add an external load, Γ>0. If the load is sufficiently small, then the bias generated by the ring will continue to produce CCW rotation (albeit at a lower rate than if the load were absent) thereby lifting the mass against gravity. In this situation, the ring settles into a steady state in which energy is systematically withdrawn from the heat bath (gas) and delivered to perform mechanical work.

## 3. Operational Modes of the Programmable Demon

More generally, the behavior of our ring depends on four parameters that we consider to be tunable: the memory cleanness δ, the bath temperature *T*, the external load Γ, and the duration of the interaction interval $\tau^{\mathrm{int}}$. All other parameters, such as the mass and density of gas particles, the length of the paddles, etc., are fixed in our model.

**Figure 5:**
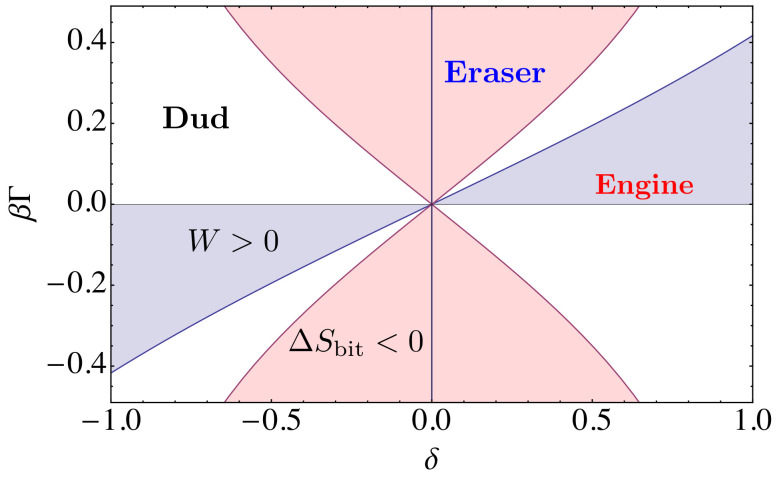
The phase diagram of the programmable Maxwell’s demon in the limit $\tau^{\mathrm{int}}\to \infty$. Here, the behavior of the ring depends only on the sequence cleanness, δ, and the external torque scaled by bath temperature, βΓ. For finite values of $\tau^{\mathrm{int}}$, the behavior of the ring depends separately on three quantities β, Γ and δ.

Depending on the values of these four parameters, the machine operates in one of three different modes—as an information engine, an information eraser or a dud. In the limit $\tau^{\mathrm{int}}\to \infty$, the model becomes analytically solvable (see Section 4.2), and its behavior is determined by the dimensionless parameters δ and βΓ, where $\beta ={\left(k_{B}T\right)}^{-1}$, as illustrated by the phase diagram shown in Figure 5. We now discuss each mode separately.

### 3.1. Engine Mode

As mentioned in Section 2.3, for δ>0 and sufficiently small Γ>0, the ring is able to convert energy drawn from the heat bath into work against the external load, thereby operating as an engine. In the limiting case δ=1, each incoming bit is matched perfectly to its reference bit, but this is no longer the case with the outgoing bits:

(5) $$\delta^{\prime }\equiv P_{\mathrm{out}}\left(\mathrm{same}\right)-P_{\mathrm{out}}\left(\mathrm{diff}\right)<1$$

More generally, when δ>0 and the ring operates in the engine mode, we have

(6) $$\delta >\delta^{\prime }>0$$

as CCW rotation tends to generate mismatches between memory bits and reference bits. Equation (6) indicates that there is greater uncertainty—less correlation with the reference bits—in the outgoing memory sequence than in the incoming sequence: $\Delta S_{L}>0$. In effect, the decrease of thermodynamic entropy associated with the continual withdrawal of energy from the heat bath is compensated by the increase of the Shannon entropy of the memory register. Our ring thus operates as an *information engine*, with a clean sequence of incoming bits serving as a thermodynamic resource that allows the system to convert heat from the bath into work against the load, without violating the second law of thermodynamics.

In the non-programmable engine of Ref. [34], an incoming sequence of 0s is converted into a mixture of 0s and 1s. It is natural to view this conversion as a process of *writing* information to the bit sequence. The outgoing pattern encodes information about the history of the ring, as outgoing 1s are correlated with CCW rotation during the corresponding interaction intervals. In the present model, by contrast, both the incoming and the outgoing sequences are mixtures of 0s and 1s. We can still view this as a process of writing information, provided this information is defined relative to the reference bits: a mismatch between an outgoing memory bit and its reference bit indicates a likelihood that CCW rotation occurred during that bit’s interaction interval. Alternatively, for the present model, we might view the incoming sequence as containing information (e.g., the binary digits of π), which is “digested” by the ring as it rectifies thermal fluctuations to generate work. Regardless of whether we interpret the ring as writing information onto a clean memory sequence or digesting information contained in that sequence, the net result is the same: when the ring acts as an engine, the outgoing bit sequence is more disordered than the incoming one, $\Delta S_{L}>0$.

### 3.2. Eraser Mode

**Figure 6:**
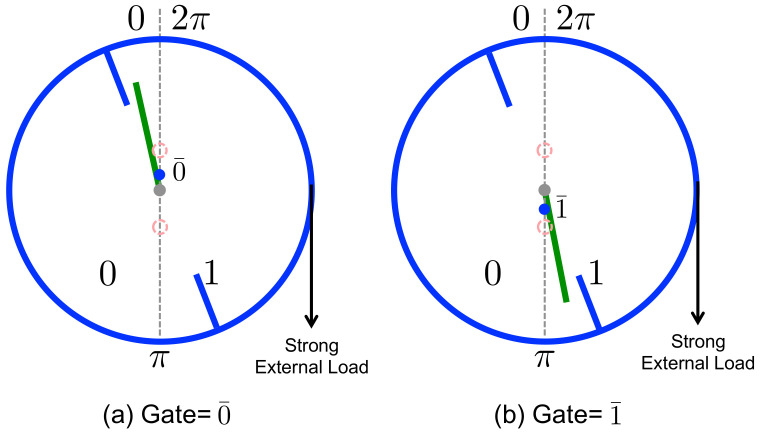
Eraser (Copier) Mode. Under a strong external load, CW rotation occurs until the bit becomes pinched between the engaging gate (shown as a blue dot situated on the gray dashed line) and a blade of the ring. The binary state of the memory bit then matches that of the reference bit.

Now let us consider what happens when: (1) the incoming bit sequence is maximally unclean (δ=0) i.e., the incoming bits are uncorrelated with the reference bits; and (2) a large mass produces a strong external load in the CW direction, Γ>0. During a given interaction interval the mass drops as far as it can, producing CW rotation of the ring until the interacting paddle (bit) is pinched between one of the blades of the ring and the rigid engaging gate associated with that paddle, as illustrated in Figure 6. If the reference bit is in state $\bar{0}$, then the engaging gate is located at θ=0 and the paddle that encodes the memory bit is forced by the CW rotation into a state $0<\theta_{B}\ll \pi$, corresponding to the binary state 0 (Figure 6a). Conversely, if the reference bit is in state $\bar{1}$, then the engaging gate is situated at θ=π and the paddle is forced into a state $\pi <\theta_{B}\ll 2\pi$, corresponding to the binary state 1 (Figure 6b). In either case, at the end of the interaction interval the memory bit matches the reference bit ($0\bar{0}$ or $1\bar{1}$).

In this mode of operation, the ring harnesses the gravitational energy of the falling mass to decrease the randomness in the bit sequence. Specifically, $\Delta S_{L}=-\log2<0$, since the outgoing bits are perfectly matched to the reference bits; see Equations (3) and (4). This decrease in the Shannon entropy of the memory bit stream is compensated by an increase in the thermodynamic entropy of the heat bath, as the energy from the falling mass is ultimately dissipated into the bath.

The model developed in Ref. [34] displays a similar mode of operation, with a falling mass converting an incoming sequence of 0s and 1s into an outgoing sequence of 0s. We referred to this mode as *Landauer’s eraser*, as it illustrates Landauer’s principle that heat must be dissipated to erase information. We use the same terminology to refer to the mode of operation just described for the present model, although *Landauer’s copier* might be more apt in this context, since the net effect is that the preprogrammed reference sequence is copied onto the memory bits.

### 3.3. Dud Mode

It is useful to think of a clean memory (δ=1) as a thermodynamic resource, just as a mass that has been lifted against gravity is a thermodynamic resource. The engine and eraser modes represent an interplay between these two resources, in which one resource is depleted to increase the other. Thus, in the engine mode, the cleanness of the memory bit stream is diminished to raise the mass against gravity, while in the eraser mode the gravitational potential energy of the mass is spent to obtain a clean memory. When the incoming bit stream is sufficiently clean and the external load (mass) is sufficiently small, the ring acts as an engine, whereas when the incoming bits are disordered and the mass is large, it acts as an eraser. For intermediate values of δ and Γ, the ring might act either in the engine mode or in the eraser mode, depending on the values of other parameters such as the interaction time $\tau^{\mathrm{int}}$ and the temperature and density of the surrounding gas.

There is also a third possibility: the mass drops while the disorder of the memory increases, $\Delta S_{L}>0$. We call this the *dud* mode, since it represents a wasteful depletion of both thermodynamic resources. This mode arises either if the incoming memory sequence contains a surplus of mismatches over correct matches, δ<0, and the load Γ>0 is not sufficiently strong to produce an even greater surplus of correct matches in the outgoing sequence—as illustrated by the white area region appearing in the second quadrant in Figure 5— or if a surplus of correct matches in the incoming sequence is not sufficient to raise the mass against gravity, while simultaneously the load Γ>0 is not sufficient to counter the tendency of the bits to randomize—as illustrated by the narrow white tongue appearing in the first quadrant in Figure 5.

In the dud mode, the Shannon entropy of the memory sequence increases, $\Delta S_{L}>0$, and the thermodynamic entropy of the surrounding gas increases, as it absorbs the energy of the falling mass.

## 4. Numerical and Analytical Results

### 4.1. Numerical Simulations

We performed numerical simulations of our contraption immersed in a dilute gas, modeling the collisions between the gas particles and the paddles and blades as Poisson processes. The probability per unit time that a gas particle strikes a particular location of a given paddle or blade was determined by the temperature *T* and density of the gas, the angular velocity of the paddle or blade, and the radial location of the point of collision. During a given interaction interval, we simulated the dynamics of the ring and the interacting bit as a sequence of events. Each event was a blade–paddle collision, a paddle–gate collision, or a collision of a gas particle with either the paddle or the blade. After each event, the angular velocity of the blade and/or paddle was appropriately updated, and the next event was generated stochastically using the Gillespie algorithm [50]. At the end of the interaction interval, the machine underwent a bit renewal, in which the old interacting bit was replaced by a new one, whose angular location $\theta_{B}$ and velocity ${\dot{\theta }}_{B}$ were assigned randomly according to the values of δ and *T*.

The degrees of freedom modeled explicitly in our simulations were the angular orientations of the demon, $\theta_{D}$, and the interacting bit, $\theta_{B}$. The steady downward motion of the stream of bits and gates was modeled implicitly as a constant interaction interval τ between bit renewal events, when the interacting bit was replaced by the next bit in line. We did not explicitly model the motion of the non-interacting bit paddles. At the moment of bit renewal, the orientation of the newly arrived interacting bit paddle was generated randomly, according to the logical state (0/1) of the bit. The interaction between the gas particles and the bit paddles or the blades of the demon was modeled as a series of discrete events, with stochastic waiting times that follow a Poisson probability rate determined by the density of the gas particles and velocity of the moving surface (i.e., the paddle or the blade). For each collision event between a gas particle and a paddle or blade, we sampled a random incoming particle velocity, as well as a random location at which the collision occurred along the paddle or blade, from consistently constructed probability distributions. Assuming elastic collisions, we computed the updated angular velocity of paddle or blade immediately after the collision.

A typical step between two events in our event-based simulation can be sketched as follows. First, compute the waiting time until the interacting bit leaves the interaction range of the demon. Then, compute the waiting time until the interacting bit collides with the demon and the waiting time until the demon collides with the gate. Then, generate a random waiting time before a collision occurs between a gas particle and the demon, and the bit, in accordance with the Poissonian probability rate mentioned in the previous paragraph. Finally, choose the event with the shortest waiting time; evolve the bit (with constant angular velocity) and the demon (with constant acceleration) until the moment of this event; and realize the change due to the event (e.g., a collision or a bit renewal). All collisions were taken to be elastic and we assumed that the blades and paddles were made of infinitely thin and rigid mesh materials to avoid secondary collisions with a gas particle. The moment of inertia of both the demon and each bit was set to 0.1. The mass of each gas particle aws also set to 0.1. The effective number density of gas particles was 1.0. The paddle for each bit took the radial range between 0.3 and 0.8 and the blade of the demon took the radial range between 0.5 and 1. The vertical dimension of both the demon and each bit was 1.0. The constant downward speed of the stream of bits was 0.1, thus the interaction interval was τ=20. Energy units were chosen such that $k_{B}T=1$.

**Figure 7:**
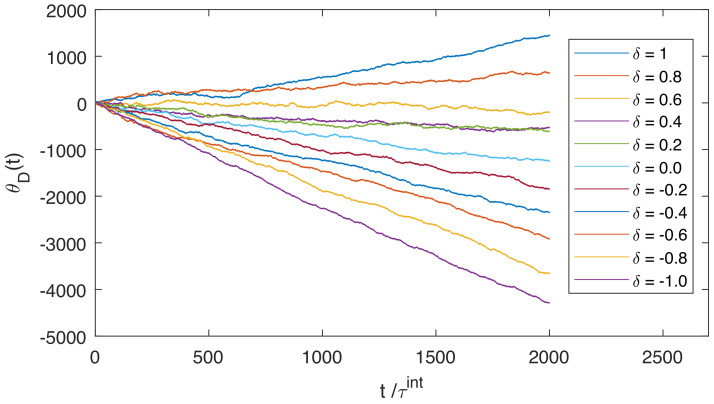
Trajectories of the ring’s angular orientation for different values of δ at fixed load $\Gamma =0.05\;k_{B}T$, with a bit renewal rate of 1 bit per 20 s ($\tau^{\mathrm{int}}=20$). For δ=1 and δ=0.8, the ring performs work against the clockwise external torque, while for the other values of δ, the external load dominates and work is dissipated into the heat bath. The cleanness of the outgoing bits for each trajectory, from δ=1 to δ=−1, is $\delta^{\prime }=0.1094,\;0.1144,\;0.1054,\;0.1244,\;0.1154,\;0.0854,\;0.1154,\;0.0864,\;0.0814,\;0.1064,\;0.0754$.

Figure 7 shows eleven angular trajectories of the angular rotation of the ring, $\theta_{D}\left(t\right)$, illustrating the engine mode and the dud mode. The simulations were performed at temperature $k_{B}T=1$ and load $\Gamma =0.05k_{B}T$, for eleven different values of the cleanness of the incoming memory bits, δ. Each simulation lasted for 2000 interaction intervals, representing 2000 incoming bits, with $\tau^{\mathrm{int}}=20$. The gates were prepared in the repeating binary sequence “...0101101011...”. In agreement with the arguments of Section 3, when δ is close to 1, the ring undergoes systematic counterclockwise rotation and the ring performs work against the external load, lifting the mass against gravity (engine mode). For less clean incoming sequences, with values δ≤0.6, the ring can no longer overcome the external torque and rotates clockwise (dud mode).

**Figure 8:**
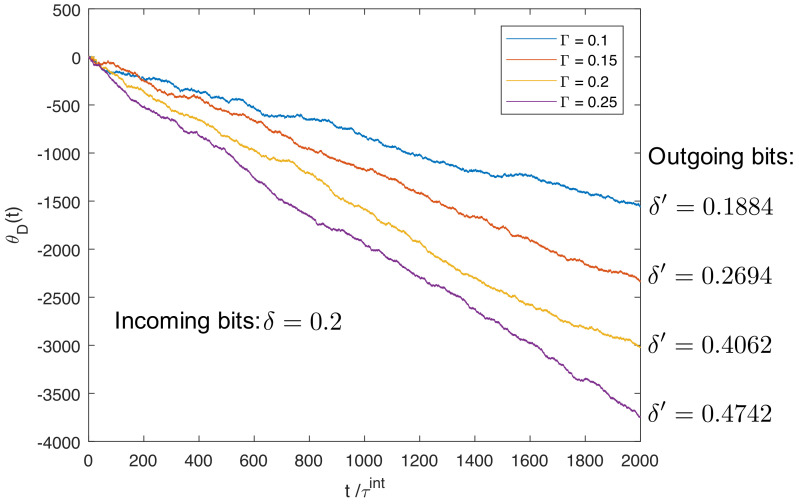
Trajectories of the ring’s angular orientation for different values of CW external torque Γ, at fixed δ=0.2. For each trajectory, the ring rotates in the CW direction and thus the energy of the falling mass is dissipated into the heat bath. With increasing external torque ($\Gamma \;=\;0.1k_{B}T,\;0.15k_{B}T,\;0.2k_{B}T,\;0.25k_{B}T$), the cleanness of the outgoing sequence of bits increases as well: $\delta^{\prime }=0.1884,\;0.2694,\;0.4062,\;0.4742$. For $\Gamma \geq 0.15k_{B}T$, we obtained $\delta^{\prime }>0.2$, hence the ring acts as an eraser, removing randomness from the incoming sequence.

To illustrate the eraser mode, Figure 8 shows four trajectories simulated as in Figure 7, except that we fix δ=0.2 and vary the external torque: $\Gamma [k_{B}T]=0.1,\;0.15,\;0.2,\;0.25$. As expected, the stronger the load is, the faster the ring rotates in the CW direction, leading to more energy dissipated into the heat bath. We found that, for $\Gamma \geq 0.15k_{B}T$, the outgoing sequence is cleaner than the incoming sequence of bits, hence the ring functions as an eraser.

### 4.2. Analytical Results for the Slow-Moving Limit

Let us now consider the limit of long interaction time $\tau^{\mathrm{int}}\to \infty$. In this limit, the behavior of the ring during one interaction interval becomes uncorrelated with its behavior in the next interval. The average work performed by the ring, *W*, and the Shannon entropy change of the memory tape, $\Delta S_{L}$, can then be computed analytically and are given by Equations (7) and (11). We now sketch the approach that is taken to obtain these results, leaving the technical details to the Appendix.

**Figure 9:**
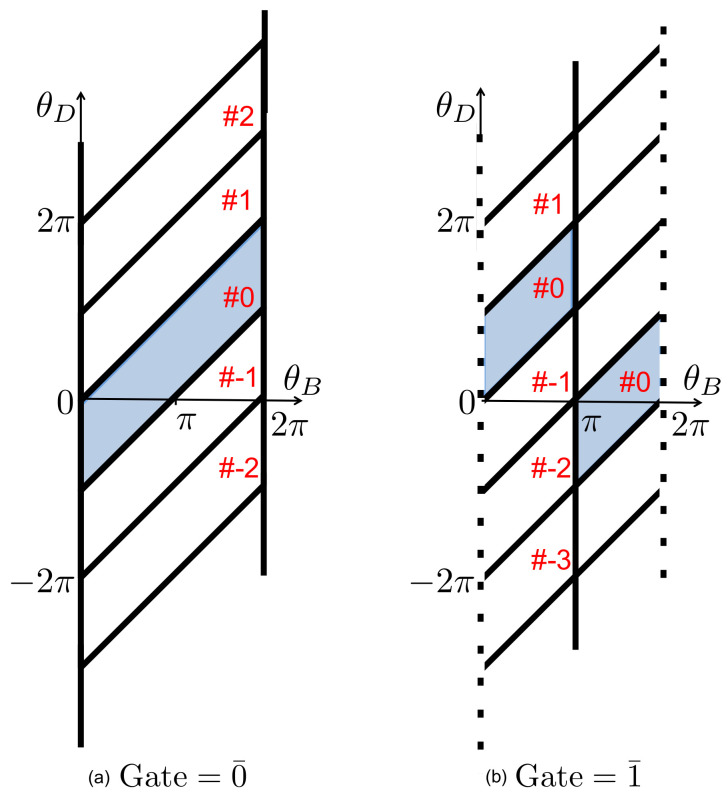
The configuration space of the ring and interacting bit. The tilted lines at $\theta_{D}-\theta_{B}=n\pi$ depict hard boundaries associated with a collision between the interacting bit paddle and either blade of the ring. The vertical solid lines correspond to the location of the engaging gate that blocks the paddle. This gate is located: at θ=0=2π when the reference bit is set to $\bar{0}$ (**a**); or at θ=π when the reference bit is set to $\bar{1}$ (**b**); The dashed lines in (**b**) represent periodic boundary conditions. The hard wall boundaries partition the configuration space into parallelogram-shaped *cells*, which are numbered as shown, with cell #0 shaded in each panel.

Letting ($\theta_{B},\theta_{D}$) denote the instantaneous configuration of the composite system—the interacting bit and the ring—we depict the relevant features of configuration space in Figure 9, with bold solid lines representing hard wall boundaries. Note that the boundary conditions depend on the state of the reference bit, $\bar{0}$ or $\bar{1}$, through the placement of the engaging gate at θ=0 or θ=π.

During a given interaction interval, the ring and interacting bit undergo random collisions with the surrounding bath particles, while the external load imposes a potential energy contribution $\Gamma \theta_{D}$ that generates a CW torque on the ring. The ring and bit are confined within a single parallelogram-shaped cell in configuration space (see Figure 9), and the composite system $\left(\theta_{B},\theta_{D}\right)$ has sufficient time to relax to equilibrium within this cell. Hence, if the composite system begins within a particular cell at the start of an interaction interval, then at the end of the interval its statistical state is given by a Boltzmann distribution restricted to that cell.

Let us suppose that during the initial interaction interval the composite system is found in one of the two shaded cells depicted in Figure 9, depending on the state of the reference bit. Let $p_{\bar{0}}^{\mathrm{eq}}\left(\theta_{B},\theta_{D}\right)$ and $p_{\bar{1}}^{\mathrm{eq}}\left(\theta_{B},\theta_{D}\right)$ denote the equilibrium distributions restricted to these two cells. The correlations between $\theta_{B}$ and $\theta_{D}$ differ in these two distributions, but if we integrate either distribution over $\theta_{B}$, then the resulting marginal equilibrium distributions for $\theta_{D}$ are identical: $p_{D}^{\mathrm{eq}}\left(\theta_{D}\right)=\int d\theta_{B}\;p_{\bar{0}}^{\mathrm{eq}}=\int d\theta_{B}\;p_{\bar{1}}^{\mathrm{eq}}$. The distribution $p_{D}^{\mathrm{eq}}$ has support in the region $-\pi \leq \theta_{D}\leq 2\pi$. In the absence of an external load, both $p_{\bar{0}}^{\mathrm{eq}}$ and $p_{\bar{1}}^{\mathrm{eq}}$ are uniform distributions within the shaded regions, and $p_{D}^{\mathrm{eq}}\left(\theta_{D}\right)$ has the shape of an isosceles trapezoid. In the opposite limit of a strong external load $\Gamma \gg k_{B}T$, $p_{D}^{\mathrm{eq}}\left(\theta_{D}\right)$ is strongly concentrated near $\theta_{D}=-\pi$ (due to the Boltzmann factor $e^{-\beta \Gamma \theta_{D}}$), as the memory bit paddle becomes pinched between one of the ring’s blades and the engaging gate.

**Figure 10:**
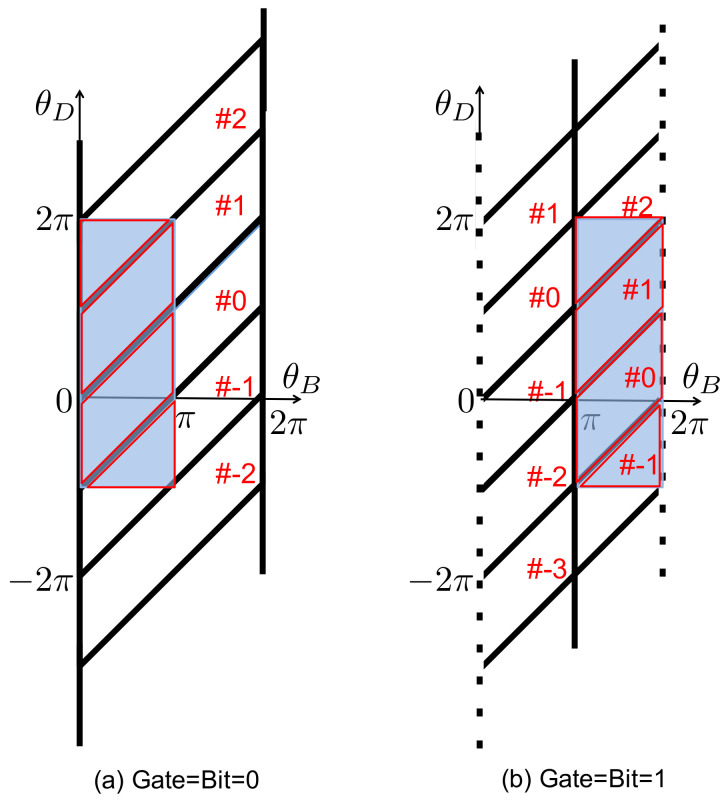
The shaded regions indicate the distribution of the composite system right after renewal, for the case when the memory bit is correctly matched with the reference bit. For purpose of illustration, we assume that just before the renewal the system was found in either one of the shaded cells shown in Figure 9, both corresponding to #0: (**a**) the new memory and reference bits are in the combined state $\left(0\bar{0}\right)$; and (**b**) the new memory and reference bits are in the combined state $\left(1\bar{1}\right)$. The marginal probability distribution of the ring’s angle, $p_{D}^{\mathrm{eq}}\left(\theta_{D}\right)$, is unaffected by the renewal mapping.

At the start of the next interaction interval, the memory and reference bits are replaced, or *renewed*, by the arrival of a new paddle and engaging gate. The location of the engaging gate now reflects the new reference bit, $\bar{0}$ or $\bar{1}$. The state of the new memory bit, *b*, either matches or mismatches the reference bit, with a probability determined by the value of δ. We can treat the configuration of the incoming memory bit as a random, uniform sample either from the range $0\leq \theta_{B}<\pi$ if b=0, or from $\pi \leq \theta_{B}<2\pi$ if b=1. This renewal process instantaneously maps the final distribution of the composite system at the end of one interaction interval, into a new initial distribution at the beginning of the next interval, as the variable $\theta_{B}$ now refers to the new memory bit rather than the old one. This mapping depends on the state of the new bit, as illustrated in Figure 10. At the start of a new interaction interval, the bit and ring configurations, $\theta_{B}$ and $\theta_{D}$, are uncorrelated.

If the machine (bit + ring) is found in cell #k during one interaction interval, and if the new, incoming memory and reference bits are correctly matched, then during the next interval it will be found in one of four possible cells, corresponding to a displacement Δk=−1,0,1 or 2, as illustrated in Figure 9 and Figure 10 for k=0. The probability distribution for Δk is determined by considering how the equilibrium distribution restricted to the initial cell (#k) is redistributed by the mapping that occurs upon bit renewal. By similar arguments, if the incoming memory and reference bits are mismatched, then the displacement is Δk=−2,−1,0 or 1.

The process then repeats itself over the next interaction interval: the probability distribution relaxes to equilibrium within each cell, and then renewal occurs when the new memory and reference bits arrive. Thus, from one interaction interval to the next, we can treat the dynamics of the ring as a discrete time random walk along a lattice of cells, with each step Δk sampled randomly from a distribution that depends on whether the incoming memory and reference bits are matched or mismatched. The net result is that Δk can range from −2 to +2, with probabilities determined by the values of δ and Γ. On average, each positive (negative) unit increment in *k* corresponds to CCW (CW) rotation of the ring by half a circle.

Following the considerations discussed above, we have computed the probability distribution for Δk analytically, and from these results we have determined the average work performed by the ring, per interaction interval (see Appendix A for details):

(7) $$W=\frac{\pi \beta \Gamma \delta -\pi \beta \Gamma \left[3\coth(\pi \beta \Gamma )+\csc h(\pi \beta \Gamma )\right]+4}{2\beta }$$

In the limit of a weak external load, Equation (7) gives

(8) $$W\approx \delta \pi \Gamma /2\;\;\;\;\;\;\;\;(0<\Gamma \ll k_{B}T)$$

and the ring acts as an engine when δ>0, in agreement with the discussion in Section 2.3.

In the opposite limit of strong external load, we get

(9) $$W\approx \left(\delta -3\right)\pi \Gamma /2\;\;\;\;\;\;\;\;\left(\Gamma \gg k_{B}T\right)$$

hence W<0, as expected. As a consistency check on Equation (7), both of the limiting cases represented by Equations (8) and (9) can be verified by directly calculating the average displacement of $\theta_{D}$ per period, resulting from the renewal mapping illustrated in Figure 9 and Figure 10.

Additionally, we can compute the fractions of bit–gate agreement and disagreement in the outgoing tape:

(10) $$P_{\mathrm{out}}\left(\mathrm{same}\right)=\frac{e^{\Gamma \pi \beta }}{e^{\Gamma \pi \beta }+1}\;,\;P_{\mathrm{out}}\left(\mathrm{diff}\right)=\frac{1}{e^{\Gamma \pi \beta }+1}.$$

In the limit of a strong external load ($\Gamma \gg k_{B}T$), virtually all outgoing bits will be forced to match the reference bits, as each bit paddle becomes pinched between then ring’s blade and the engaging gate (see Section 3.2). Per interaction period, the change of the Shannon entropy of the memory tape with respect to the gate is

(11) $$\begin{array}{cc} \Delta S_{L} & =\frac{1-\delta }{2}\log\left(\frac{1-\delta }{2}\right)+\frac{1+\delta }{2}\log\left(\frac{1+\delta }{2}\right)\\ & -\frac{e^{\pi \beta \Gamma }}{e^{\pi \beta \Gamma }+1}\log\left(\frac{e^{\pi \beta \Gamma }}{e^{\pi \beta \Gamma }+1}\right)-\frac{1}{e^{\pi \beta \Gamma }+1}\log\left(\frac{1}{e^{\pi \beta \Gamma }+1}\right) \end{array}$$

where recall that the variable L=B Exy G is the Boolean equality between the state of the bit and the state of the gate (see Section 2.2).

Combining Equations (7) and (11), we obtain (see Appendix B for details)

(12) $$\Delta S_{L}-\frac{W}{k_{B}T}=\;D_{KL}\left[P_{\mathrm{in}}|P_{\mathrm{out}}\right]+\frac{\pi \beta \Gamma }{\tanh(\pi \beta \Gamma /2)}-2$$

where $D_{KL}\geq 0$ is the Kullback–Leibler divergence [51] between the incoming and outgoing bit distributions. Since x/tanh(x)>1 for all x≠0, Equation (12) implies

(13) $$k_{B}\Delta S_{L}-\frac{W}{T}\geq 0$$

which is a strict inequality when Γ≠0. Because the work *W* is equal to the average energy extracted from the heat bath, per bit, the term −W/T represents the net change in the thermodynamic entropy of bath. As a result, Equation (13) can be viewed as a statement of the second law of thermodynamics: the sum of the entropy changes of the bit stream and heat bath must be non-negative. Notice that this interpretation relies on treating the information content of the bit stream (multiplied by $k_{B}$) as a genuine thermodynamic entropy, on par with the Clausius entropy.

Equation (13) suggests natural definitions of the machine’s thermodynamic efficiency in both the engine and the eraser mode. When the ring functions as an eraser, we have

(14) $$W<k_{B}T\Delta S_{L}<0$$

and the efficiency is defined as

(15) $$\eta_{\mathrm{eraser}}=\frac{k_{B}T\Delta S_{L}}{W}<1$$

When the ring functions as an engine,

(16) $$k_{B}T\Delta S_{L}>W>0$$

and the efficiency is defined as

(17) $$\eta_{\mathrm{engine}}=\frac{W}{k_{B}T\Delta S_{L}}<1$$

When the ring functions in the dud mode, $W<0<k_{B}T\Delta S_{L}$.

**Figure 11:**
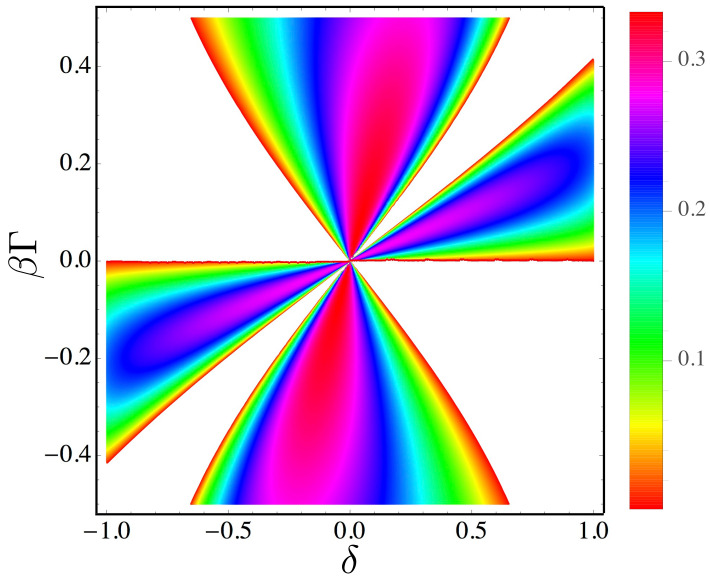
Efficiency plot of the programmable demon, obtained analytically in the limit $\tau^{\mathrm{int}}\to \infty$. Since efficiency is defined only for the eraser and engine modes, the dud region is left blank.

In Figure 11, we plot the thermodynamic efficiency over the phase diagram of the machine. By definition η>0 within the regions corresponding to the engine and eraser modes, but η drops to zero at the boundaries of these regions, where the ring becomes a dud. For example, a point on the boundary of the engine mode, with δ,β,Γ>0, represents a stalled state. Here, the ring generates just enough CCW torque to match the CW torque exerted by the external load (hence W=0), nevertheless there is a positive rate of entropy generation in the bit stream ($\Delta S_{L}>0$). If the load Γ is decreased by a small amount, then the ring will produce a slight CCW rotation, resulting in an engine with very low efficiency.

### 4.3. Second Law of Thermodynamics in the Slow Moving Limit

We obtained Equation (13) from our exact solution of the dynamics in the slow-moving limit, but the result has the character of a generalized, information-theoretic second law of thermodynamics (as already mentioned), and its validity may extend to finite values of $\tau^{\mathrm{int}}$. While it is difficult to establish this validity from first principles, we can make some progress by ignoring correlations (of any sort) from one interval to the next, as we do in the following statistical treatment in which the variables *B* and *G* are treated as *information-bearing degrees of freedom* [52].

At the start of an interaction interval, let $P_{BG}^{in}\left(b,g\right)$ denote the joint probability to find the memory bit in state b∈{0,1} and the reference gate in state $g\in \{\bar{0},\bar{1}\}$, and let $P_{B}^{in}\left(b\right)$ and $P_{G}^{in}\left(g\right)$ denote the corresponding marginal distributions. Let $S_{BG}$, $S_{B}$ and $S_{G}$ denote the Shannon entropies of these distributions.

Then,

(18) $$S_{BG}^{in}=S_{B}^{in}+S_{G}^{in}-I_{BG}^{in}$$

where

(19) $$I_{BG}^{in}=\sum_{b,g}P_{BG}^{in}\log\frac{P_{BG}^{in}}{P_{B}^{in}P_{G}^{in}}\geq 0$$

is the *mutual information* [53] between the bit and gate states. Defining similar quantities for the outgoing states, the net change in the combined entropy over one interaction interval is

(20) $$\begin{array}{cc} \Delta S_{BG} & =\Delta S_{B}+\Delta S_{G}-\Delta I_{BG}\\ & =\Delta S_{B}-\Delta I_{BG} \end{array}$$

where $\Delta S_{BG}=S_{BG}^{out}-S_{BG}^{in}$, etc. Since the state of the gate remains fixed, we have $\Delta S_{G}=0$, whereas both $S_{B}$ and $I_{BG}$ typically change during the interaction interval.

We have used the variables *B* and *G* to specify the combined state of a memory and reference bit, but we could equally well specify this state using the variables *L* and *G*, leading to

(21) $$\Delta S_{BG}=\Delta S_{LG}=\Delta S_{L}-\Delta I_{LG}$$

where $\Delta S_{LG}$, $\Delta S_{L}$ and $I_{LG}$ are defined as above, but with *L* in place of *B*.

The Hamiltonian analysis of Ref. [29] (see in particular Equation (47) therein) suggests that the change in the Shannon entropy of the information-bearing degrees of freedom *B* and *G* obeys a generalized second law of thermodynamics: $W/k_{B}T\leq \Delta S_{BG}$. Combining with Equation (21) gives us

(22) $$\frac{W}{k_{B}T}\leq \Delta S_{L}-\Delta I_{LG}=\Delta S_{L}-I_{LG}^{out}$$

Here, we have used our assumption that incoming mismatches are statistically uncorrelated with the state of the gate (Section 2.2) to set $I_{LG}^{in}=0$. Since mutual information is non-negative, Equation (22) immediately implies Equation (13), but note that Equation (22) provides a stronger bound than Equation (13). In effect, if correlations develop between the reference gate *G* and the logical state *L*, then these correlations represent an “unused” information-thermodynamic resource. In the slow-moving limit, these correlations vanish since the demon and bit fully equilibrate, hence Equation (22) reduces to Equation (13) in that limit.

## 5. Our Machine As a Feedback-Controlled Device

In previous sections, we have presented our model as an autonomous system, whose various components (paddles, gas particles, etc.) evolve without external interference. With a slight modification our model can serve to illustrate a *non-autonomous* device: a machine that is manipulated via measurement and feedback. In this non-autonomous interpretation, the ring can again operate as an engine that lifts a mass against gravity, as we describe in Section 5.1. We then show how the inequality given by Equation (22) for the autonomous case can be translated into an inequality that applies to non-autonomous measurement and feedback (Equation (26)). Finally, in Section 5.3, we show how our model can be modified to act as a non-autonomous device that uses the energy of a dropping mass to write a desired target sequence to a stream of bits.

### 5.1. Feedback-Controlled Engine

**Figure 12:**
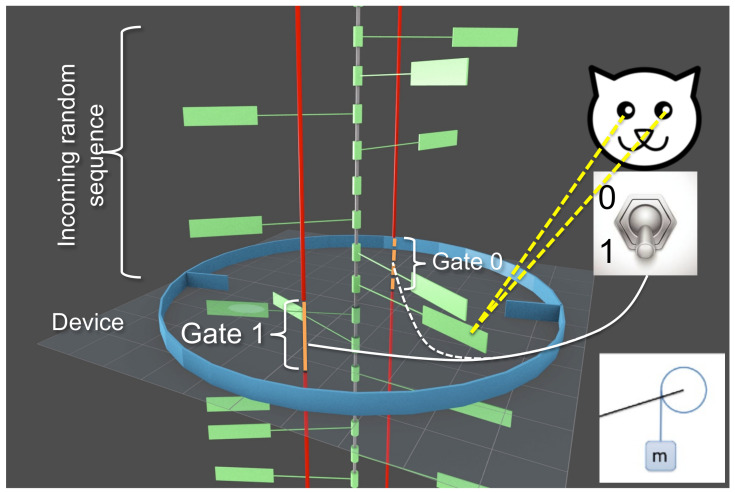
A non-autonomous version of our model. The snapshot is taken right at the time of bit renewal. The agent observes the state of the new bit (state 1) and simultaneously sets the gate $\bar{1}$ to be effective. Thus, the new bit can switch state only through the unblocked gate $\bar{0}$. In this illustration, the agent’s measurement is faithful and thus the ring is able to work in the engine mode.

Consider a setup that is essentially the same as that described in Section 2, but without the sequence of rigid reference gates (the blue L-shaped bars in Figure 3). In their place is a single, switchable gate that can be set to block either one of the two gaps (in the red blocking bars) positioned at the vertical location of the ring. We say that the gate is in the $\bar{0}$ state when it blocks the gap at θ=0, and in the $\bar{1}$ state when it blocks the gap at θ=π; the latter case is depicted in Figure 12.

Throughout this section, we assume that the incoming bits arrive in a fully randomized sequence, with 0s and 1s distributed equally. We introduce an external agent who performs measurement and feedback on these bits (see Figure 12). The agent observes each new bit as it arrives, and at the moment of bit renewal (when the incoming bit becomes the interacting bit) the agent sets the switchable gate accordingly: if it observes the incoming bit to be in state 0 (or 1), it sets the switchable gate to state $\bar{0}$ (or $\bar{1}$).

If the agent performs error-free measurements, faithfully identifying the state of each incoming bit, then from the perspective of the ring the situation is equivalent to the case δ=1 analyzed in Section 2. Namely, the blocked gate is matched with the state of the incoming bit so as to produce, during each interaction interval, a statistical bias in favor of CCW rotation. In the long run, this bias can cause a small mass to be lifted against gravity, systematically extracting energy from the heat bath and thereby reducing its entropy. Since (by assumption) the incoming bits arrive in a fully randomized sequence, the decrease in the entropy of the bath cannot be “paid for” by increasing the Shannon entropy of the bits. Rather, the model illustrates how an external agent, by performing measurement and feedback, can rectify fluctuations to produce an apparent violation of the second law of thermodynamics. Of course there is no real violation, as the physical nature of the agent is not being taken into account—similar to Maxwell, we have effectively inserted a “magical creature” into our model.

We further generalize this scenario to include the possibility of measurement errors. For each incoming bit, let ϵ denote the probability that the agent misidentifies the bit state and therefore blocks the “wrong” gate. This situation is equivalent to the one analyzed in Section 3.1, with δ=1−2ϵ. For sufficiently small error rate ϵ and load Γ, the machine may still lift the mass against gravity, despite the measurement errors.

The non-autonomous model described in this section is similar to Maxwell’s original thought experiment, and even more so to the Szilard engine [35], in which an agent determines whether a gas particle is within the left or right half of a box, then appropriately attaches a mass that can be lifted by the expansion of the single-particle gas. In our model, the “expansion” of a bit paddle from the half-circle to the full circle during each interaction interval plays the role of the expansion of the single-particle gas in the Szilard model. Note, however, that, in the case of the Szilard engine, the same gas particle is recycled from one iteration of the measurement-and-feedback process to the next, whereas our model uses a sequence of “gas particles” (incoming bits) that can act as a memory register. This allows our model to act not only as an engine but also as a device that writes information, as we discuss in Section 5.3.

### 5.2. The Second Law of Thermodynamics with Feedback Control

We have noted the equivalence between the measurement-and-feedback scenario described in Section 5.1 (with error rate ϵ) and the autonomous engine of Section 3.1 (with δ=1−2ϵ). Let us use this equivalence to obtain a second law inequality for the measurement-and-feedback process.

As above, let $P_{BG}^{in}\left(b,g\right)$ denote the joint probability distribution describing the initial state of the bit and blocked gate—just after the agent has measured the bit and set the gate accordingly. During the interaction interval, $0<t<\tau^{\mathrm{int}}$, the machine operates autonomously, hence (see Section 4.3)

(23) $$\frac{W}{k_{B}T}\leq \Delta S_{BG}=\Delta S_{G}+\Delta S_{B}-\Delta I_{BG}$$

Since the gate state *G* does not change during the interaction interval, $\Delta S_{G}=0$. In addition, since the fully randomized incoming bit stream contains equal populations of 0s and 1s, the same will be true (by symmetry) of the outgoing bit stream, hence $S_{B}^{in}=S_{B}^{out}=\log2$, and $\Delta S_{B}=0$. We thus get

(24) $$\frac{W}{k_{B}T}\leq -\Delta I_{BG}=I_{BG}^{in}-I_{BG}^{out}$$

The initial mutual information is simply the information gained by the measurement process:

(25) $$I_{BG}^{in}=I_{meas}=\log2+\left(1-\epsilon \right)\log\left(1-\epsilon \right)+\epsilon \log\epsilon$$

The final mutual information quantifies the degree to which *B* and *G* remain correlated at the end of the interval; we refer to this value as the *residual* information: $I_{res}=I_{BG}^{out}$. We thus have

(26) $$\frac{W}{k_{B}T}\leq I_{meas}-I_{res},$$

i.e., the extracted work *W* is bounded by the amount of information gathered during the measurement, minus the amount “left over” at the end of the interval. Hence, the gathered information is a thermodynamic resource, and the difference $I_{meas}-I_{res}$ represents the amount of that resource that is consumed, per interaction interval. Since $I_{res}\geq 0$, Equation (26) immediately implies the weaker bound

(27) $$\frac{W}{k_{B}T}\leq I_{meas}.$$

Equation (27) was originally derived within the framework of stochastic thermodynamics by Sagawa and Ueda in Refs. [27,28], and Equation (26) was subsequently obtained by the same authors in Refs. [30,54]. We also note that the net change in the mutual information between the bit and the gate, $\Delta I_{BG}$, can be interpreted as the integrated *information flow*, within the bipartite approach developed by Horowitz and Esposito [31]. This information flow is negative (hence $I_{meas}-I_{res}>0$), as information is consumed to extract energy to lift the mass.

### 5.3. Feedback-Controlled Information Recorder

In the eraser mode discussed in Section 3.2, our autonomous machine removes randomness from the incoming bit stream, replacing it with a preprogrammed sequence encoded in the reference gates. In the present context of an externally manipulated machine, let us imagine that the agent desires to write a particular target sequence, e.g., 011010⋯, to the bit stream. The agent does not perform measurements on the incoming bits, but as each bit arrives the agent sets the switchable gate to match the corresponding element of the target sequence. Then, as in Section 3.2, the CW torque produced by the gravitational pull of the mass produces a tendency to set the state of the interacting bit to match the desired target value, through the “pinching” mechanism illustrate in Figure 6. The fidelity of the writing process increases with the torque Γ generated by the gravitational force on the mass, and the energy of the dropping mass is dissipated into the heat bath.

## 6. Concluding Remarks

In this paper, we present a model of a programmable, mechanical Maxwell’s demon that can be interpreted either as an autonomous device, as described in Section 2, Section 3 and Section 4, or as a non-autonomous device manipulated by external measurement and feedback control, as in Section 5. For these distinct interpretations, we have obtained distinct forms of the second law of thermodynamics, represented by Equations (22) and (26). While these results have been obtained within the specific context of our model, it would be useful to investigate whether they point to more general thermodynamic laws, in situations involving both autonomous and non-autonomous (i.e., feedback-controlled) devices. For instance, as indicated in Section 5.2, the inequalities given by Equations (26) and (27) have been obtained previously under assumptions of bipartite, Markovian dynamics [27,28,30,31,54]. By contrast, we have obtained these results within a Newtonian model of colliding particles and paddles, which suggests that they might be derived more generally within a classical, Hamiltonian framework.

Additionally, we have obtained analytical results for the work delivered by our device, Equation (7), and the change in the Shannon entropy of the bits, Equation (11), in the limit of a slowly-moving stream of bits, $\tau^{\mathrm{int}}\to \infty$. For finite $\tau^{\mathrm{int}}$, the interactions between the bits and the demon may induce statistical correlations among the outgoing bits. Such correlations, which could in principle act as a thermodynamic resource, have not been considered in our analysis. It would be interesting to investigate how these correlations might affect the inequalities that we have derived. 

## Appendix A. Work Delivered Per Interaction Interval

To compute the mean work that our device delivers to lift a mass against gravity, we must compute the mean angular displacement of the ring per interaction interval. This displacement can be determined by considering the transition from the end of an interaction interval (see Figure 9) to the beginning of the next interaction interval (see Figure 10). Recall that, during a particular interaction interval, the composite system is confined within a single cell in $\left(\theta_{B},\theta_{D}\right)$-space, as illustrated by the shaded region in Figure 9a. At the moment of bit renewal the value of $\theta_{B}$ changes suddenly, hence the system may find itself in a different cell immediately after bit renewal, as illustrated in Figure 10a. We characterize this transition by the change Δk in the cell index. At a coarse-grained level, the evolution of the system from one interval to the next becomes a random walk of discrete jumps in *k*-space.

Here, we compute the probability of the jumps conditioned on the agreement or disagreement between the state of the bit and its corresponding gate. At the moment of bit renewal, the state of the system can remain in the same cell (Δk=0), can jump up or down by one cell (Δk=±1), or can jump up or down by two cells (Δk=±2). Note however that the value Δk=+2 is possible only if the incoming bit matches the incoming gate (as in Figure 10), and the value Δk=−2 can occur only if the bit and gate are mismatched. To illustrate how to compute the probabilities of these various events, let us imagine that, immediately after renewal, both the incoming bit and its reference gate are in the 0 state. Then, the probability distribution for the system is partitioned among the four shaded regions appearing in Figure 10a, each of which corresponds to a particular value of Δk, and the distribution within each of these cells is inherited from the equilibrium distribution just prior to bit renewal (Figure 9). By integrating over the distribution within each region, and then summing over all possible combinations of incoming bit and gate, we obtain the following results.

When the incoming bit agrees with its gate (e.g., bit = 1 and gate = $\bar{1}$ or bit = 0 and gate = $\bar{0}$), we have

(A1) $$P_{+2}^{\mathrm{same}}\equiv P_{+2}^{0,\bar{0}}=P_{+2}^{1,\bar{1}}=\frac{\pi \beta \Gamma +e^{\pi \beta \Gamma }\left(\pi \beta \Gamma -2\right)+2}{\pi \beta \Gamma {\left(e^{\pi \beta \Gamma }-1\right)}^{2}\left(e^{\pi \beta \Gamma }+1\right)}$$

(A2) $$P_{+1}^{\mathrm{same}}\equiv P_{+1}^{0,\bar{0}}=P_{+1}^{1,\bar{1}}=\frac{e^{\pi \beta \Gamma }\left[\pi \beta \Gamma \left(e^{\pi \beta \Gamma }-3\right)+2\right]-2}{\pi \beta \Gamma {\left(e^{\pi \beta \Gamma }-1\right)}^{2}\left(e^{\pi \beta \Gamma }+1\right)}$$

(A3) $$P_{\mathrm{stay}}^{\mathrm{same}}\equiv P_{\mathrm{stay}}^{0,\bar{0}}=P_{\mathrm{stay}}^{1,\bar{1}}=\frac{e^{\pi \beta \Gamma }\left[\pi \beta \Gamma +e^{\pi \beta \Gamma }\left(-3\pi \beta \Gamma +2e^{\pi \beta \Gamma }-2\right)\right]}{\pi \beta \Gamma {\left(e^{\pi \beta \Gamma }-1\right)}^{2}\left(e^{\pi \beta \Gamma }+1\right)}$$

(A4) $$P_{-1}^{\mathrm{same}}\equiv P_{-1}^{0,\bar{0}}=P_{-1}^{1,\bar{1}}=\frac{e^{2\pi \beta \Gamma }\left[\pi \beta \Gamma +e^{\pi \beta \Gamma }\left(\pi \beta \Gamma -2\right)+2\right]}{\pi \beta \Gamma {\left(e^{\pi \beta \Gamma }-1\right)}^{2}\left(e^{\pi \beta \Gamma }+1\right)}$$

(A5) $$P_{-2}^{\mathrm{same}}\equiv P_{-2}^{0,\bar{0}}=P_{-2}^{1,\bar{1}}=0$$

If the incoming bit mismatches its gate, we find

(A6) $$P_{+2}^{\mathrm{diff}}\equiv P_{+2}^{0,\bar{1}}=P_{+2}^{1,\bar{0}}=0$$

(A7) $$P_{+1}^{\mathrm{diff}}\equiv P_{+1}^{0,\bar{1}}=P_{+1}^{1,\bar{0}}=\frac{\pi \beta \Gamma +e^{\pi \beta \Gamma }\left(\pi \beta \Gamma -2\right)+2}{\pi \beta \Gamma {\left(e^{\pi \beta \Gamma }-1\right)}^{2}\left(e^{\pi \beta \Gamma }+1\right)}$$

(A8) $$P_{\mathrm{stay}}^{\mathrm{diff}}\equiv P_{\mathrm{stay}}^{0,\bar{1}}=P_{\mathrm{stay}}^{1,\bar{0}}=\frac{e^{\pi \beta \Gamma }\left[\pi \beta \Gamma \left(e^{\pi \beta \Gamma }-3\right)+2\right]-2}{\pi \beta \Gamma {\left(e^{\pi \beta \Gamma }-1\right)}^{2}\left(e^{\pi \beta \Gamma }+1\right)}$$

(A9) $$P_{-1}^{\mathrm{diff}}\equiv P_{-1}^{0,\bar{1}}=P_{-1}^{1,\bar{0}}=\frac{e^{\pi \beta \Gamma }\left[\pi \beta \Gamma +e^{\pi \beta \Gamma }\left(-3\pi \beta \Gamma +2e^{\pi \beta \Gamma }-2\right)\right]}{\pi \beta \Gamma {\left(e^{\pi \beta \Gamma }-1\right)}^{2}\left(e^{\pi \beta \Gamma }+1\right)}$$

(A10) $$P_{-2}^{\mathrm{diff}}\equiv P_{-2}^{0,\bar{1}}=P_{-2}^{1,\bar{0}}=\frac{e^{2\pi \beta \Gamma }\left[\pi \beta \Gamma +e^{\pi \beta \Gamma }\left(\pi \beta \Gamma -2\right)+2\right]}{\pi \beta \Gamma {\left(e^{\pi \beta \Gamma }-1\right)}^{2}\left(e^{\pi \beta \Gamma }+1\right)}$$

The net probability to take a step Δk at the moment of bit renewal is then given by

(A11) $$R_{\Delta k}=P_{\mathrm{in}}\left(\mathrm{same}\right)\cdot P_{\Delta k}^{\mathrm{same}}+P_{\mathrm{in}}\left(\mathrm{diff}\right)\cdot P_{\Delta k}^{\mathrm{diff}}=\frac{1+\delta }{2}P_{\Delta k}^{\mathrm{same}}+\frac{1-\delta }{2}P_{\Delta k}^{\mathrm{diff}}$$

Over a long observation time, i.e., many interaction intervals, these discrete jumps in *k*-space produce average rotations by amounts $\Delta \theta_{D}=-2\pi ,\;-\pi ,\;0,\;\pi ,\;2\pi$, per time interval $\tau^{\mathrm{bit}}$. The average work delivered per interval is simply the average rotation of the demon per interval multiplied, by the external torque:

(A12) $$\begin{array}{cc} W & =2\pi \Gamma \cdot R_{+2}+\pi \Gamma \cdot R_{+1}-\pi \Gamma \cdot R_{-1}-2\pi \Gamma \cdot R_{-2}\\ & =\frac{\pi \beta \Gamma \delta -\pi \beta \Gamma \left[3\coth(\pi \beta \Gamma )+\csc h(\pi \beta \Gamma )\right]+4}{2\beta } \end{array}$$

## Appendix B. Compatibility with the Second Law of Thermodynamics

Here, we show that our analytical solution of the programmable demon in the slow-moving limit obeys the second law of thermodynamics.

For convenience, define x≡δ and $y\equiv \left(e^{\lambda }-1\right)/\left(e^{\lambda }+1\right)$, where λ≡πβΓ. Then, Equation (11) becomes

(A13) $$\Delta S_{L}=\frac{1-x}{2}\ln\frac{1-x}{2}+\frac{1+x}{2}\ln\frac{1+x}{2}-\left(\frac{1-y}{2}\ln\frac{1-y}{2}+\frac{1+y}{2}\ln\frac{1+y}{2}\right)$$

and the dimensionless work done per bit (Equation (7)) is

(A14) $$\begin{array}{c} \beta W=\frac{\lambda x}{2}-\frac{\lambda }{2}\left[3\coth(\lambda )+\mathrm{csch}(\lambda )\right]+2 \end{array}$$

(A15) $$\begin{array}{c} \;\;\;\;=\frac{x}{2}\ln\frac{1+y}{1-y}+2-\frac{1}{2}\left(\frac{2}{y}+y\right)\ln\frac{1+y}{1-y} \end{array}$$

Taking the difference, we get

(A16) $$\begin{array}{cc} \Delta S_{L}-\beta W & =-2+\frac{1}{y}\ln\frac{1+y}{1-y}+\frac{x}{2}\ln\frac{\left(1+x)(1-y\right)}{\left(1-x)(1+y\right)}+\frac{1}{2}\ln\frac{\left(1+x)(1-x\right)}{\left(1-y)(1+y\right)} \end{array}$$

(A17) $$\begin{array}{c} =\frac{x+1}{2}\ln\frac{(1+x)/2}{(1+y)/2}+\frac{1-x}{2}\ln\frac{(1-x)/2}{(1-y)/2}+\frac{1}{y}\ln\frac{1+y}{1-y}-2 \end{array}$$

where x∈[−1,+1] and y∈(−1,+1). Note also that $P_{in}\left(\mathrm{same}/\mathrm{diff}\right)=\left(1\pm x\right)/2$ (see Equation (1)) and $P_{out}\left(\mathrm{same}/\mathrm{diff}\right)=\left(1\pm y\right)/2$ (see Equation (10)), which allows us to rewrite the above expression as

(A18) $$\Delta S_{L}-\beta W=D_{KL}\left[P_{in}|P_{out}\right]+\frac{1}{y}\ln\frac{1+y}{1-y}-2$$

where $D_{KL}\geq 0$ is the Kullback–Leibler divergence [51] between the incoming and outgoing bit distributions.

Next, we show that

(A19) $$\frac{1}{y}\ln\frac{1+y}{1-y}-2\geq 0$$

by expanding the logarithm as an infinite series:

(A20) $$\begin{array}{cc} \frac{1}{y}\ln\frac{1+y}{1-y}-2 & =\frac{1}{y}\left(\sum_{n=0}^{\infty }\frac{{\left(-1\right)}^{n}y^{n+1}}{n+1}+\sum_{n=0}^{\infty }\frac{y^{n+1}}{n+1}\right)-2 \end{array}$$

(A21) $$\begin{array}{c} =\frac{1}{y}\sum_{n=0}^{\infty }\frac{2y^{2n+1}}{2n+1}-2 \end{array}$$

(A22) $$\begin{array}{c} =\sum_{n=1}^{\infty }\frac{2y^{2n}}{2n+1}\geq 0 \end{array}$$

where the equality is achieved when y=0. Alternatively, we can rewrite the left side in terms of λ:

(A23) $$\frac{1}{y}\ln\frac{1+y}{1-y}-2=\frac{\lambda }{\tanh(\lambda /2)}-2=2\left(\frac{\lambda /2}{\tanh(\lambda /2)}-1\right)\geq 0$$

where the last inequality follows since |a|≥|tanha| for any real *a*.

We thus confirm that in the slow-moving limit, our Maxwell’s demon satisfies $\Delta S_{L}-\beta W\geq 0$, where the equality is achieved only when the external force is absent and the incoming sequence is totally random.
